# Clinical prognosis evaluation of alpha-fetoprotein-positive gastric cancer: comprehensive analysis and development of a novel nomogram for survival prediction

**DOI:** 10.3389/fonc.2025.1598337

**Published:** 2025-05-23

**Authors:** Fei Zuo, Yunyan Tan, Wenjun Mao, Zhaoqing Tang, Tianping Luo

**Affiliations:** ^1^ Department of General Surgery, Changzhou Traditional Chinese Medicine Hospital, Affiliated Hospital of Nanjing University of Chinese Medicine, Changzhou, China; ^2^ Department of General Surgery, Zhongshan Hospital, Fudan University, Shanghai, China

**Keywords:** alpha-fetoprotein, gastric cancer, prognosis, metastasis, nomogram

## Abstract

**Background:**

Alpha-fetoprotein (AFP) is an established biomarker for liver cancer, but its role in gastric cancer (GC) remains unclear. This study evaluated AFP’s prognostic value in GC and developed a survival prediction model incorporating AFP and other clinical factors.

**Methods:**

We analyzed 766 GC patients from Changzhou Traditional Chinese Medicine Hospital, categorizing them as AFP-positive (>20 ng/mL) or AFP-negative (≤20 ng/mL). Kaplan-Meier and Cox regression analyses assessed the association between AFP levels and overall survival (OS). A nomogram based on identified prognostic factors was created and evaluated using ROC curves, calibration curves, and decision curve analysis (DCA).

**Results:**

Among 766 gastric cancer (GC) patients, 3.3% (n=25) exhibited elevated AFP levels (>20 ng/mL). The AFP-positive group demonstrated significantly more aggressive clinicopathological features, including larger tumor size (*p* < 0.05), deeper invasion (higher T-stage), increased lymph node metastasis (higher N-stage), and higher rates of distant metastasis (*p* = 0.035). Survival analysis revealed markedly worse outcomes for AFP-positive patients (Log-rank *P* < 0.001), with a 68% higher mortality risk (unadjusted HR =1.68, 95% CI: 1.27–2.23). Multivariate Cox regression confirmed AFP positivity as an independent prognostic factor (adjusted HR = 1.8, 95% CI: 1.03–3.14, *p* =0.04), alongside T4-stage, N3-stage, and distant metastasis. A prognostic nomogram integrating AFP levels and TNM staging achieved superior predictive accuracy (AUCs: 0.80–0.84) compared to TNM staging alone (AUCs: 0.70–0.74) across 1-, 3-, and 5-year survival. Calibration and decision curve analyses further validated the model’s clinical utility, supporting its role in risk stratification and treatment planning.

**Conclusions:**

AFP is a significant independent prognostic factor in gastric cancer, and its inclusion in a multivariate model enhances survival prediction. The prognostic nomogram developed in this study offers clinicians a valuable tool for predicting patient outcomes and guiding treatment decisions. Further validation and prospective studies are necessary to confirm the model’s clinical applicability.

## Introduction

Gastric cancer constitutes a major global health challenge, particularly within Asia, where its incidence and mortality rates are considerably elevated (1). As reported by the World Health Organization (WHO), gastric cancer ranks as the fifth most common cancer worldwide, maintaining a consistently high mortality rate (2). Despite notable advancements in surgical techniques, radiotherapy, chemotherapy, and targeted therapies, the early manifestations of gastric cancer often remain insidious (3). This frequently results in diagnoses at advanced stages, at which point treatment efficacy is significantly diminished. The elevated mortality rate and associated treatment complexities render gastric cancer a pressing public health concern, necessitating urgent action. Therefore, the implementation of early screening procedures, timely diagnoses, and effective prognostic evaluations is essential for enhancing patient survival rates (4).

Alpha-fetoprotein (AFP) is identified as a glycoprotein predominantly secreted by the fetal liver and gastrointestinal tract, with concentrations typically remaining low in adults (5). Elevated AFP levels are frequently associated with hepatocellular carcinoma, specific germ cell tumors, and gastric hepatoid adenocarcinoma (GHA), among other malignancies (6). In recent years, AFP’s potential diagnostic and prognostic utility in gastric cancer has garnered increasing attention, particularly within subtypes such as gastric hepatoid adenocarcinoma (GHA), where elevated levels are significantly correlated with tumor development, progression, and prognosis. Elevated AFP levels may not only correlate with tumor occurrence but could also reflect underlying tumor behavior, encompassing factors such as invasiveness, metastatic potential, and treatment response. Consequently, AFP is regarded as a significant biomarker in gastric cancer research (7).

While numerous studies underscore the potential of AFP in the context of gastric cancer, existing literature predominantly focuses on its correlation with specific clinical and pathological features. Research indicates that elevated AFP is closely associated with advanced tumor stage, increased tumor size, lymph node involvement, and distant metastasis. Patients with AFP-positive status frequently present with larger tumors, higher rates of lymph node metastasis, and more frequent liver metastasis, all of which are commonly linked to a poorer prognosis. However, systematic studies specifically addressing AFP-positive gastric cancer patients are sparse, with many characterized by limitations such as small sample sizes and patient heterogeneity (8). As a result, despite preliminary research efforts, the absence of comprehensive prognostic models constrains the effective clinical application of AFP as a biomarker in gastric cancer.

This study seeks to retrospectively analyze the clinical and pathological characteristics of AFP-positive gastric cancer patients and investigate the relationship between AFP levels and clinical outcomes. By integrating AFP with other critical clinical and pathological factors, this research aims to develop a prognostic model using multifactorial analysis, with the objective of providing a more precise reference for prognostic evaluation and individualized treatment in clinical practice.

## Materials and methods

### Study population

A retrospective analysis was conducted on 1,100 gastric cancer patients treated at Changzhou Traditional Chinese Medicine Hospital, an affiliate of Nanjing University of Chinese Medicine. Of these, 766 patients meeting the inclusion criteria were selected for comprehensive analysis. D2 radical gastrectomy was performed on all patients, except for 18 individuals with gastric cancer liver metastasis who underwent simultaneous hepatogastric lesion resection. The inclusion criteria were as follows (1): a confirmed diagnosis of gastric adenocarcinoma (2), comprehensive clinical and pathological data, and (3) available preoperative AFP measurement results. Patients with underlying liver diseases (e.g., cirrhosis, hepatic fibrosis, or hepatocellular carcinoma) or other conditions known to elevate AFP levels were excluded, as these conditions could bias the AFP’s role as a gastric cancer biomarker. The protocol of this study was approved by the institutional ethical board of Changzhou Traditional Chinese Medicine Hospital. The study was conducted in accordance with the Declaration of Helsinki (as revised in 2013).

### AFP measurement and grouping

Preoperative serum AFP levels were determined using a chemiluminescent immunoassay, with a normal reference range of 0-20 ng/mL. Patients were classified into two groups based on serum AFP level (8) (1): AFP-positive (serum AFP >20 ng/mL) and (2) AFP-negative (serum AFP ≤ 20 ng/mL). This threshold was selected in accordance with established clinical practices for determining AFP positivity in gastric cancer.

### Data collection and follow-up

Clinical and pathological data were retrospectively obtained from medical records. The collected data encompassed demographic information (age, sex), tumor characteristics (size), and laboratory results (AFP, CEA, and CA19-9 levels). Pathological staging information, including T-stage (tumor depth), N-stage (lymph node involvement), and M-stage (distant metastasis), was cataloged. Follow-up procedures included outpatient visits, telephone calls, emails, and letters. The final follow-up was conducted in December 2019. Survival time was defined as the interval from surgery to either the last follow-up date or the date of death, whichever occurred first. Patients who were lost to follow-up were not included in the survival analysis.

### Statistical analysis

Descriptive statistics were employed to summarize the baseline characteristics of the study population. Continuous variables were expressed as means with standard deviations (SD), whereas categorical variables were presented as frequencies and percentages. Differences between the AFP-positive and AFP-negative groups were analyzed using statistical tests, such as Student’s t-test for continuous variables and chi-squared tests for categorical variables. Kaplan-Meier survival analysis was employed to evaluate overall survival (OS) between the two groups. Survival curves were generated, and differences were assessed using the log-rank test. Hazard ratios (HR) and 95% confidence intervals (CI) were calculated to quantify the mortality risk associated with AFP positivity. Univariate and multivariate Cox proportional hazards regression analyses were utilized to identify significant prognostic factors. Key variables consisted of AFP levels, T-stage, N-stage, tumor size, and other relevant clinical parameters. Predictors identified as significant in univariate analysis were subsequently included in a multivariate Cox regression model to determine independent prognostic factors.

### Prognostic model construction

A nomogram was constructed based on findings from the multivariate Cox regression analysis. It incorporated significant independent prognostic factors, including AFP levels, T-stage, N-stage, and tumor size, to project individual patient survival probabilities at various time points. To ensure the robustness and generalizability of the model, the study population was randomly divided into a modeling cohort and a validation cohort in a ratio of 7:3. The modeling cohort was utilized for constructing the nomogram, while the validation cohort was used to evaluate its performance. This nomogram functions as an efficient tool for clinicians to estimate patient outcomes and facilitate clinical decision-making.

### Model evaluation

The nomogram’s performance was assessed using receiver operating characteristic (ROC) curves, with the area under the curve (AUC) providing a measure of its discriminative ability. Calibration curves were utilized to compare predicted survival probabilities with observed outcomes, thus evaluating the nomogram’s predictive accuracy. Decision curve analysis (DCA) provided insights into the clinical utility of the nomogram by comparing the potential benefits of its application versus treating all or no patients.

Statistical analyses were conducted using R software (version 4.2). A *p*-value of <0.05 was considered statistically significant for all analyses.

## Results

### Patient characteristics

The study cohort comprised 766 gastric cancer patients, among whom 25 (3.3%) were identified as AFP-positive (serum AFP > 20 ng/mL). The mean patient age was 62.9 ± 11.6 years, predominantly male (60.8%). In **Table 1**, baseline characteristics of both AFP-positive and AFP-negative groups are detailed. Significantly higher occurrences of advanced tumor stages, including larger tumor size, greater invasion (higher T-stage), and more frequent lymph node involvement (higher N-stage), were observed in the AFP-positive group compared to the AFP-negative group (all *p* < 0.05). Additionally, liver and distant metastases were more common in the AFP-positive group (*p* = 0.263 and *p* = 0.035, respectively). To further validate the consistency of AFP’s prognostic value, we performed stratified analyses by sex (male/female), age (≤65/>65 years), TNM stage (I-II/III-IV), and liver metastasis status (yes/no). While some subgroups did not reach statistical significance due to limited sample size (all interaction *P*-values >0.05), The forest plot (**Supplementary Figure S1**) demonstrated good homogeneity across subgroups, supporting the stability of AFP’s prognostic value regardless of patient characteristics.

**Table 1:** Analysis of the sociodemographic characteristics of the research object.

Variables	Total (n = 766)	AFP Negative (n = 741)	AFP Positive (n = 25)	*P*
Age (years)	62.9 ± 11.6	62.8 ± 11.7	65.5 ± 11.0	0.268
Tumor Size (cm)	4.80 ± 2.35	4.75 ± 2.30	5.90 ± 2.70	0.032*
CA19-9 (U/mL)	83.84 ± 418.39	79.86 ± 411.95	201.83 ± 576.39	0.152
CEA (ng/mL)	5.86 ± 16.39	5.51 ± 15.52	16.10 ± 32.07	0.113
Number of lymph node	21.66 ± 9.80	21.75 ± 9.84	19.00 ± 8.21	0.168
Lymph node (mm)	5.61 ± 7.16	5.56 ± 7.20	7.08 ± 5.80	0.297
Survival Time (days)	1395.74 ± 925.38	1414.51 ± 923.66	839.24 ± 810.06	0.002*
Sex, n (%)				
Female	300 (39.2)	290 (39.1)	10 (40.0)	0.918
Male	466 (60.8)	451 (60.9)	15 (60.0)	
T, n (%)				0.024*
1	127 (16.57)	127 (17.14)	0 (0.00)	
2	92 (12.01)	91 (12.28)	1 (4.00)	
3	197 (25.71)	188 (25.37)	9 (36.00)	
4	350 (45.69)	335 (45.21)	15 (60.00)	
N, n (%)				0.047*
0	249 (32.51)	246 (33.20)	3 (12.00)	
1	138 (18.02)	132 (17.81)	6 (24.00)	
2	135 (17.62)	132 (17.81)	3 (12.00)	
3	244 (31.85)	231 (31.17)	13 (52.00)	
M, n (%)				0.035*
0	743 (97.00)	721 (97.30)	22 (88.00)	
1	23 (3.00)	20 (2.70)	3 (12.00)	
Histology, n (%)				
AD	500 (65.3)	486 (65.6)	14 (56.0)	0.263
MAD	110 (14.4)	106 (14.3)	4 (16.0)	
SRCC	80 (10.4)	77 (10.4)	3 (12.0)	
Other	76 (9.9)	72 (9.7)	4 (16.0)	
Liver Metastases, n (%)				
Absent	748 (97.65)	726 (97.98)	22 (88.00)	0.263
Present	18 (2.35)	15 (2.02)	3 (12.00)	

t, t-test; χ², Chi-square test; -, Fisher exact; *P<0.05.

SD, standard deviation.

### Survival analysis based on AFP levels

**Figure 1:**
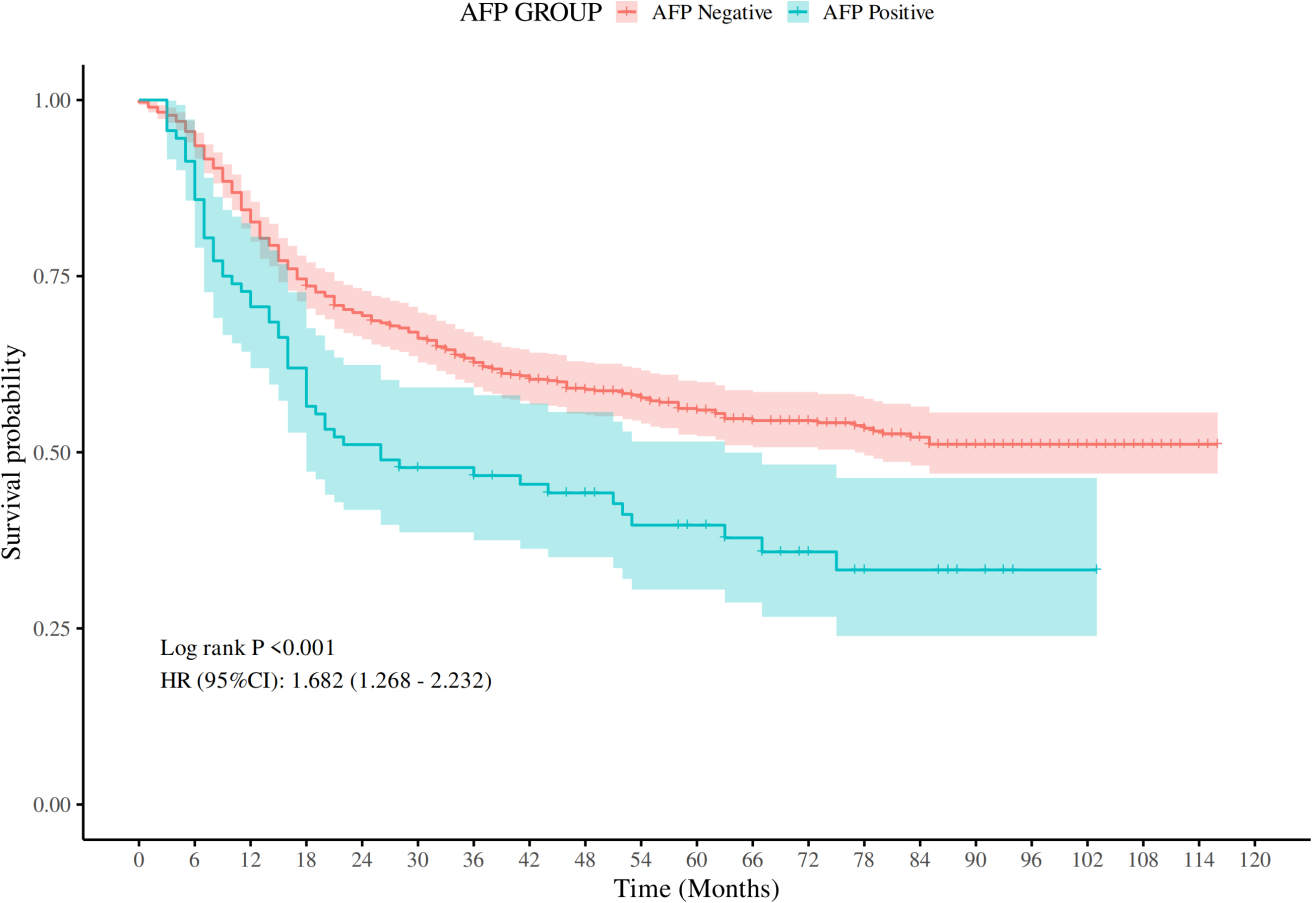
Comparison of OS rates between AFP-positive GC and AFP-negative GC (P < 0.001).

For the purposes of survival analysis, patients were segregated into AFP-positive and AFP-negative groups. Kaplan-Meier survival curves demonstrated a significant difference in OS between the two groups. Those in the AFP-positive group had significantly poorer outcomes (Log-rank *P* < 0.001). The hazard ratio (HR) for the AFP-positive cohort, compared to the AFP-negative cohort, was calculated to be 1.68 (95% confidence interval (CI: 1.27–2.23), suggesting that elevated AFP levels correlate with a 68% increased mortality risk (**Figure 1**).

### Univariate and multivariate Cox regression analysis

Univariate Cox regression analysis indicated that several clinical and pathological factors held significant associations with OS, such as AFP levels, T-stage, N-stage, tumor size, and distant metastasis occurrence. Notably, elevated AFP levels within the AFP-positive group correlated with a significantly poorer prognosis (HR = 2.36, 95% CI: 1.37–4.05, *p* < 0.001). Multivariate Cox regression analysis, accounting for potential confounders, confirmed AFP positivity as an independent prognostic factor (adjusted HR = 1.8, 95% CI: 1.03–3.14, *p* =0.04), alongside T4-stage, N3-stage, and distant metastasis (**Table 2**). Prior to modeling, multicollinearity was assessed through generalized variance inflation factors (GVIF), with all adjusted GVIF^(1/(2*Df)) values below 1.68 (T-stage:1.06; N-stage:1.22; metastasis:1.02), confirming variable independence (**Supplementary Table S1**). This unadjusted association was further validated in multivariable Cox analysis, where AFP positivity remained significant after adjusting for T/N/M-stage(HR = 1.8, 95% CI: 1.03–3.14, *p* = 0.04), along with T4-stage (HR = 3.31, 95% CI: 1.79–6.12, *p* < 0.01), N3-stage (HR = 2.35, 95% CI: 1.49–3.71, *p* < 0.01), and distant metastasis (HR = 1.85, 95% CI: 1.08–3.16, *p* =0.03).

**Table 2:** COX regression analysis of risk factors for DM in training cohort.

Factor	Category	Univariate analysis			Multivariate analysis		
		HR	95%CI	*P*	HR	95%CI	*P*
Age (years)		1.02	1.01–1.03	0.01*	1.01	0.99–1.02	0.08
Sex	Female	1.00			–		
	Male	1.04	0.99–1.09	0.16	–		
AFP Custom (ng/mL)	AFP-	1.00			1.00		
	AFP+	2.36	1.37–4.05	<0.001*	1.80	1.03–3.14	0.04*
T Stage	T1	1.00			1.00		
	T2	1.32	0.56–3.08	0.53	1.01	0.43–2.38	0.98
	T3	3.82	2.11–6.91	<0.01*	2.55	1.38–4.74	<0.001*
	T4	6.91	3.92–12.19	<0.01*	3.31	1.79–6.12	<0.01*
N Stage	N0	1.00			1.00		
	N1	1.58	0.94–2.64	0.08	1.21	0.71–2.05	0.48
	N2	3.17	2.12–4.73	<0.01*	1.91	1.24–2.93	<0.001*
	N3	5.35	3.72–7.69	<0.01*	2.35	1.49–3.71	<0.01*
M Stage	M0	1.00			–		
	M1	3.50	2.10–5.85	<0.01*	1.85	1.08–3.16	0.03*
Histology	AD	1.00			–		
	MAD	0.95	0.85–1.05	0.37	–		
	SRCC	0.95	0.71–1.26	0.72	–		
	Other	0.88	0.73–1.03	0.13	–		
Liver Metastases	Absent	1.00			–		
	Present	2.45	1.42–4.22	<0.001*	–		
Tumor Size (cm)		1.15	1.08–1.22	<0.01*	1.07	0.99–1.15	0.08
CA19-9 (U/mL)		1.01	1.01–1.01	<0.01*	–		
CEA (ng/mL)		1.01	1.01–1.01	0.04*	–		
Number of lymph node		0.99	0.98–1.01	0.29	–		
Lymph node (mm)		1.05	1.04–1.06	<0.01*	1.02	1.01–1.04	0.01*

Multivariable HR adjusted for T/N/M-stage, tumor size. *P<0.05.

### Prognostic model construction and evaluation

**Figure 2:**
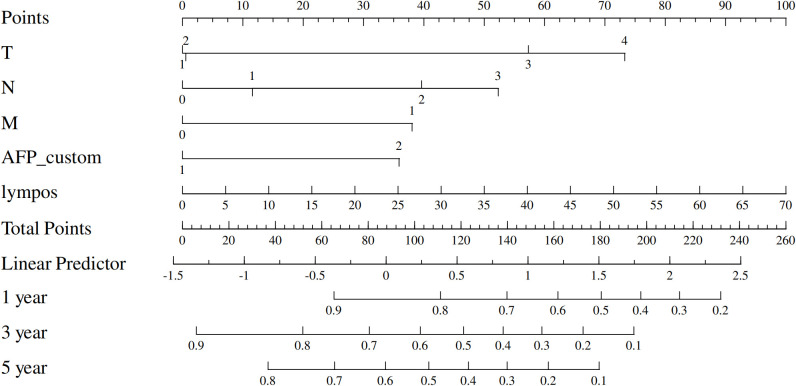
Prognostic nomogram for predicting 1-, 3-, and 5-year overall survival in gastric cancer patients.

**Figure 3:**
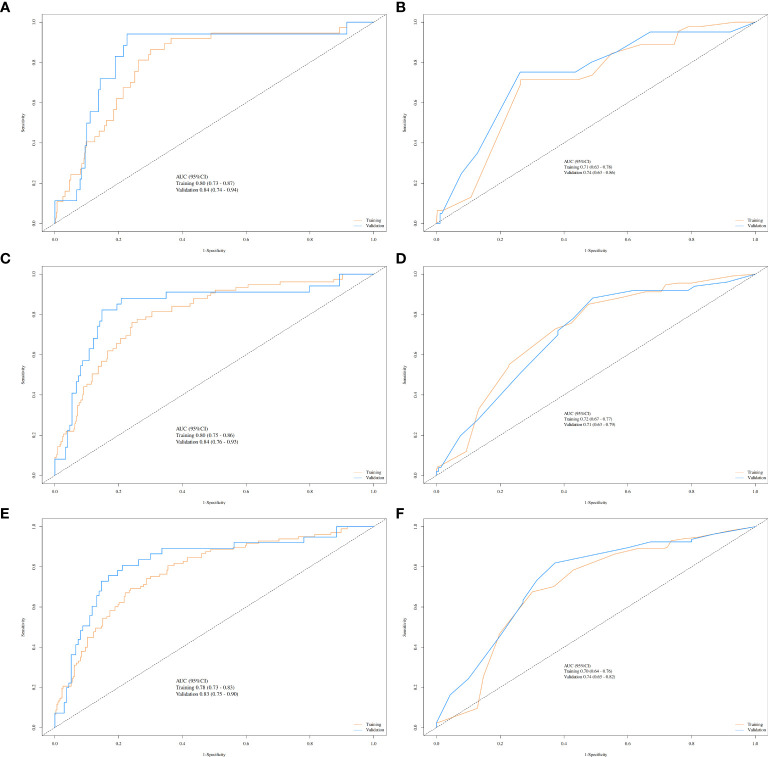
ROC curves comparing the nomogram and TNM staging system for predicting survival in gastric cancer. Nomogram performance (yellow lines: training cohort; blue lines: validation cohort) for 1-year **(A)**, 3-year **(C)**, and 5-year **(E)** survival. TNM staging performance (yellow lines: training cohort; blue lines: validation cohort) for 1-year **(B)**, 3-year **(D)**, and 5-year **(F)** survival.

**Figure 4:**
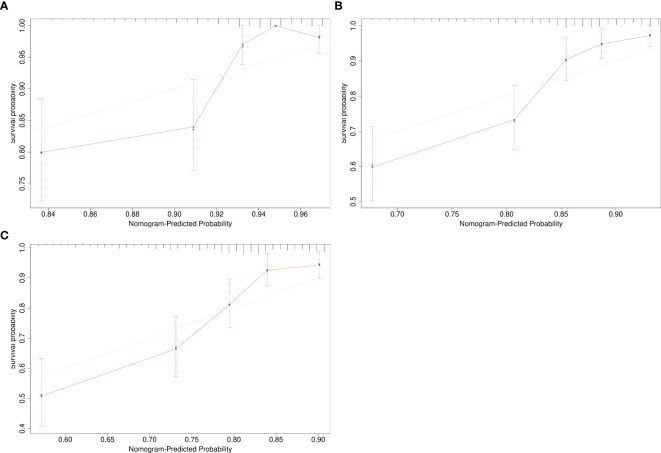
Calibration curves assessing the agreement between nomogram-predicted and observed survival probabilities. **(A)** 1-year survival; **(B)** 3-year survival; **(C)** 5-year survival.

**Figure 5:**
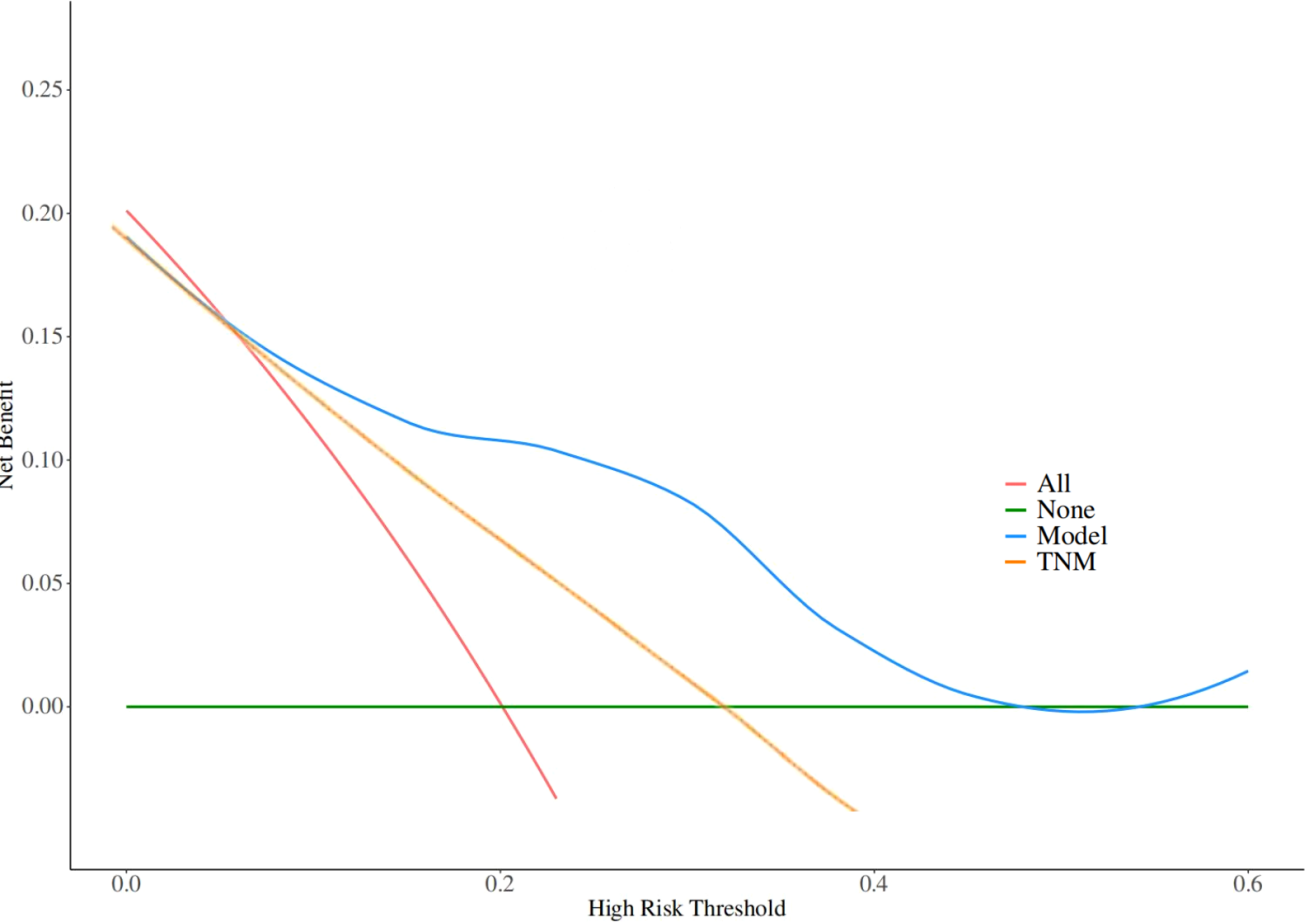
Decision curve analysis (DCA) evaluating the clinical utility of the nomogram across threshold probabilities. The nomogram (blue line) demonstrates significantly higher net benefit compared to the TNM staging system (yellow line) and the 'treat-all' (red lines) or 'treat-none' strategies (green lines).

Given the significant prognostic factors identified through multivariate analysis, a nomogram was crafted to predict 1-year, 3-year, and 5-year OS probabilities for AFP-positive gastric cancer patients (**Figure 2**). Incorporating AFP level, T-stage, N-stage, and distant metastasis, the nomogram demonstrated superior discriminatory power compared to the conventional TNM staging system. Specifically, the area under the curve (AUC) for 1-year survival in the training cohort was 0.80 (95% CI: 0.73–0.87) (**Figure 3A**)versus 0.71 (0.63–0.78) (**Figure 3B**) for TNM staging, and 0.84 (0.74–0.94) (**Figure 3A**) versus 0.74 (0.63–0.86) (**Figure 3B**) in the validation cohort. Similarly, for 3-year survival, the nomogram achieved AUCs of 0.80 (0.75–0.86) and 0.84 (0.76–0.93) in the training and validation cohorts(**Figure 3C**), respectively, outperforming TNM staging (training: 0.72 [0.67–0.77]; validation: 0.71 [0.63–0.79]) (**Figure 3D**). At 5 years, the nomogram maintained robust performance with AUCs of 0.78 (0.73–0.83) and 0.83 (0.75–0.90) (**Figure 3E**), compared to 0.70 (0.64–0.76) and 0.74 (0.65–0.82) for TNM staging (**Figure 3F**). Calibration curves showed excellent agreement between predicted and observed survival probabilities (**Figures 4A-C**), while DCA further highlighted the nomogram’s clinical utility. Across threshold probabilities, the nomogram provided significantly higher net benefits than both TNM staging and the “treat-all” or “treat-none” strategies, underscoring its value in guiding personalized treatment decisions (**Figure 5**).

## Discussion

While alpha-fetoprotein (AFP) is a well-recognized biomarker for liver cancer (9), its role in gastric cancer (GC) remains less explored. This study provides substantial evidence supporting the prognostic value of AFP in gastric cancer, particularly for patients who are AFP-positive (10). Our findings reveal a correlation between AFP-positive gastric cancer (AFPGC) and more aggressive disease characteristics, coupled with significantly worse OS. Specifically, AFP-positive patients displayed a hazard ratio (HR) of 1.68 (95% CI: 1.27–2.23), indicating a 68% increase in mortality risk compared to AFP-negative patients. These results concur with existing literature (11), which have demonstrated an association between AFP positivity and advanced tumor stages, alongside poor prognosis in various malignancies.

A central finding of our study is the link between AFP positivity and advanced clinical as well as pathological features, including larger tumor size, deeper invasion (T-stage), more frequent lymph node metastasis (N-stage), and presence of distant metastasis. Such attributes suggest that AFP-positive GC epitomizes a more aggressive disease variant. In line with previous reports, we observed a notable association between AFP-positive GC and liver metastasis; 12.0% of AFP-positive patients exhibited liver metastasis, a figure lower than the 33%-72% reported elsewhere (12). This variation might result from our inclusion criteria, focusing solely on surgically treated patients, thereby excluding more advanced, untreated cases. Nonetheless, our findings still support the notion that AFP-positive gastric cancer harbors a propensity for liver metastasis, contributing to its poorer prognosis.

Elevated AFP levels in GC may reflect both the tumor’s biological behavior and its interaction with the liver. Typically, AFP is produced in the fetal liver and yolk sac, with levels remaining low in adults (13). Reactivation of AFP expression can occur in certain tumors, especially those with hepatocellular traits (6). A potential biological mechanism underlying elevated AFP levels in gastric cancer involves hepatic differentiation or hepatocellular-like differentiation of gastric tumor cells (10). The stomach and liver originate from the primitive foregut during embryogenesis, suggesting that some gastric tumors may acquire hepatocellular features, culminating in AFP production (14). This process is more likely in gastric cancers manifesting liver metastasis, as tumor cells may acquire hepatic features during liver invasion (15). In our study, AFP-positive patients exhibited significantly larger tumor sizes (5.90 ± 2.70 cm *vs*. 4.75 ± 2.30 cm, *P* = 0.032), supporting the hypothesis that AFP-positive tumors tend to be more aggressive (16). Larger tumors have a higher propensity to metastasize, particularly to the liver, consistent with the higher rate of liver metastasis observed in AFP-positive patients (17). Additionally, increased AFP production may reflect hepatic differentiation presence in tumor cells, potentially enhancing their metastatic potential (18). This mechanism proposes that AFP might not only serve as a prognostic biomarker but also offer insights into the underlying biology of gastric cancer (6). Biological aggressiveness of alpha-fetoprotein (AFP)-positive gastric cancer The prognostic implications of elevated AFP levels are considerable (19). In our study, AFP-positive gastric cancer patients demonstrated significantly lower survival rates at 1, 3, and 5 years compared to AFP-negative patients. The 5-year survival rates for AFP-positive and AFP-negative patients were 28% and 51%, respectively, underscoring the strong association between AFP positivity and poor long-term outcomes (10). This finding implies that AFP could serve not only as a prognostic biomarker but also as a potential therapeutic target. Future research should explore whether targeting pathways related to AFP production or hepatic differentiation in GC offers novel therapeutic avenues (15).

Our study demonstrates AFP’s strong prognostic value in gastric cancer, particularly regarding metastatic propensity. This observation aligns with emerging understanding of AFP-producing tumors’ unique biology. The staining intensity of CXCR4 and SDF-1 in cancer cells was significantly higher in intestinal-type than in diffuse-type gastric cancer. Furthermore, intestinal-type cancer cells that permeated the vascular or lymphatic channels as well as liver and lymph node metastases showed strong CXCR4 and SDF-1 staining (20). Additionally, AFP acts as a co-chaperone of heat shock protein 90 (HSP90), stabilizing oncoproteins c-MYC and c-MET, thereby facilitating tumor progression in liver and gastric cancers (21). The molecular mechanism underlying AFP’s role in GC involves activation of the Wnt signaling pathway, leading to increased cell growth and aggression (22).

Our study also devised a nomogram to predict survival in AFP-positive gastric cancer patients, incorporating AFP levels along with T-stage, N-stage, and distant metastasis presence. The nomogram demonstrated robust discriminatory ability, evidenced by AUC values of 0.80 for 1-year survival in the training cohort and 0.84 in the validation cohort. Calibration curves further validated the model, confirming its accuracy in predicting actual patient outcomes. DCA also indicated that the nomogram offered a net benefit across various threshold probabilities, thus making it a valuable tool for clinical decision-making (23). This nomogram could assist clinicians in predicting individual patient survival probabilities and facilitate more informed treatment decisions, particularly for those with AFP-positive gastric cancer (24, 25).

Despite its contributions, this study has limitations. The retrospective design may carry inherent biases, including issues with data completeness and selection bias. Although the study involved a large cohort, its single-center nature limits the external validity of the findings. Future multicenter studies are necessary to validate these results and assess the nomogram’s generalizability. Additionally, AFP testing variability across laboratories may affect result reproducibility. Standardization of AFP measurement techniques is crucial for broader clinical application.

## Conclusion

In conclusion, AFP represents a significant prognostic marker in gastric cancer, and its inclusion in multivariate models enhances survival prediction. The nomogram developed in this study offers clinicians a valuable tool for predicting patient outcomes and facilitating informed treatment decisions.

## Data Availability

The original contributions presented in the study are included in the article/**Supplementary Material**. Further inquiries can be directed to the corresponding author.
