# Supplementary material for: Clinical prognosis evaluation of alpha-fetoprotein-positive gastric cancer: comprehensive analysis and development of a novel nomogram for survival prediction
Source: Front Oncol. 2025 May 23;15:1598337. doi: 10.3389/fonc.2025.1598337 (PMC12141012; doi:10.3389/fonc.2025.1598337)
Supplement: Supplementary file 1 [file Table1.doc]

**Supplementary Table S1 Multicollinearity evaluation of candidate predictors via generalized VIF**

| Variable | GVIF | Df | Adjusted GVIF (GVIF^(1/(2*Df))) | Collinearity Judgment |  |
| --- | --- | --- | --- | --- | --- |
|  |
| T | 1.408 | 3 | 1.059 | No collinearity |  |
| N | 3.34 | 3 | 1.223 | Mild collinearity (acceptable) |  |
| M | 1.033 | 1 | 1.016 | No collinearity |  |
| lympos | 2.819 | 1 | 1.679 | Mild collinearity (acceptable) |  |
| AFP_custom | 1.026 | 1 | 1.013 | No collinearity |  |
