# Supplementary figures and images for: Clinical prognosis evaluation of alpha-fetoprotein-positive gastric cancer: comprehensive analysis and development of a novel nomogram for survival prediction
Source: Front Oncol. 2025 May 23;15:1598337. doi: 10.3389/fonc.2025.1598337 (PMC12141012; doi:10.3389/fonc.2025.1598337)

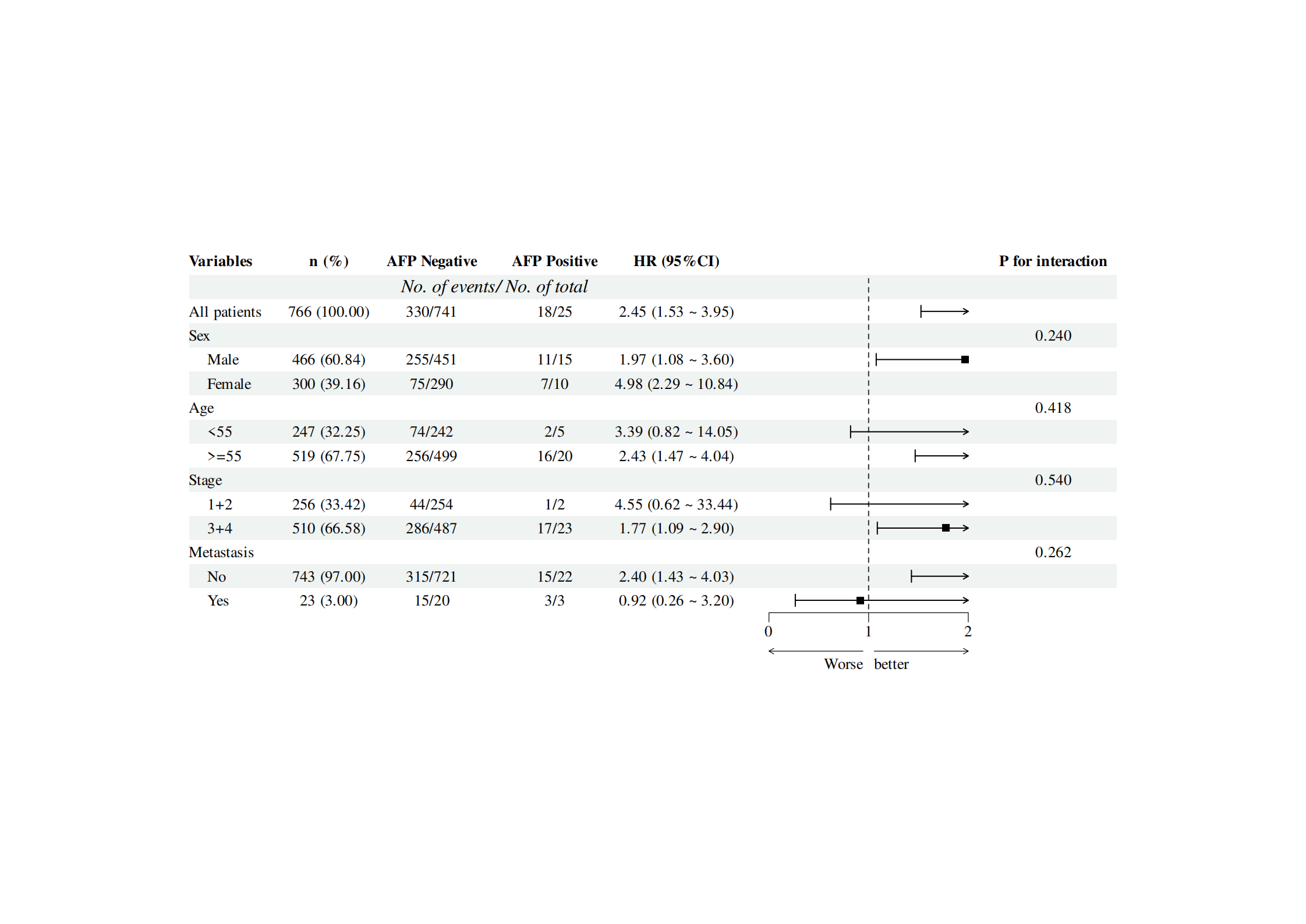

Supplement: Supplementary file 2 [file Image1.tif]
